# Neurogranin Regulates Metaplasticity

**DOI:** 10.3389/fnmol.2019.00322

**Published:** 2020-01-24

**Authors:** Ling Zhong, Nashaat Z. Gerges

**Affiliations:** Department of Cell Biology, Neurobiology, and Anatomy, Medical College of Wisconsin, Milwaukee, WI, United States

**Keywords:** LTP, LTD, synaptic plasticity, calmodulin, neurogranin, CaMKII

## Abstract

Long-term potentiation (LTP) and long-term depression (LTD) are two major forms of synaptic plasticity that are widely accepted as cellular mechanisms involved in learning and memory. Metaplasticity is a process whereby modifications in synaptic processes shift the threshold for subsequent plasticity. While metaplasticity has been functionally observed, its molecular basis is not well understood. Here, we report that neurogranin (Ng) regulates metaplasticity by shifting the threshold toward potentiation, i.e., increasing Ng in hippocampal neurons lowers the threshold for LTP and augments the threshold for LTD. We also show that Ng does not change the ultrastructural localization of calmodulin (CaM)-dependent protein Kinase II (CaMKII) or calcineurin, critical enzymes for the induction of LTP and LTD, respectively. Interestingly, while CaMKII concentrates close to the plasma membrane, calcineurin concentrates away from the plasma membrane. These data, along with the previous observation showing Ng targets CaM closer to the plasma membrane, suggesting that shifting the localization of CaM within the dendritic spines and closer to the plasma membrane, where there is more CaMKII, may be favoring the activation of CaMKII vs. that of calcineurin. Thus, the regulation of CaM localization/targeting within dendritic spines by Ng may provide a mechanistic basis for the regulation of metaplasticity.

## Introduction

Synaptic connections modify their strength and undergo continuous remodeling in response to neuronal activity. This process, known as synaptic plasticity, is widely thought as the cellular basis underlying learning and memory (Alkon and Nelson, 1990; Bliss and Collingridge, 1993; Chen and Tonegawa, 1997; Baudry, 1998; Elgersma and Silva, 1999; Kandel et al., 2000; Martin et al., 2000; Maren, 2001; Benfenati, 2007). Long-term potentiation (LTP) and long-term depression (LTD) are the two best-characterized forms of synaptic plasticity. At excitatory synapses in the CA1 area, both LTP and LTD share a common pathway for their induction and coexist in a tight balance (Lisman, 1989; Mizuno et al., 2001). They both require synaptic activation of postsynaptic N-methyl-D-aspartate (NMDA) receptors (NMDAR). Activation of NMDARs elevates the intracellular Ca^2+^ concentration within a specific range. Two Ca^2+^/calmodulin (CaM)-dependent enzymes are essential for the bidirectional balance between LTP and LTD. Ca^2+^/CaM-dependent protein phosphatase calcineurin, which is activated at low Ca^2+^/CaM concentration, is required for LTD (Klee et al., 1979; Hubbard and Klee, 1987; Mulkey et al., 1994; Torii et al., 1995; Zeng et al., 2001; Yasuda et al., 2003). On the other hand, Ca^2+^/CaM-dependent protein Kinase II (CaMKII), which is activated at high Ca^2+^/CaM concentration, is required for LTP (Miller and Kennedy, 1985; Meyer et al., 1992; Silva et al., 1992; Giese et al., 1998; Hudmon and Schulman, 2002; Lisman et al., 2002; Kennedy et al., 2005; Shifman et al., 2006; Shonesy et al., 2014). Thus, CaM availability plays a crucial role in setting the balance between LTP and LTD.

One of the most abundant postsynaptic proteins that bind to CaM is neurogranin (Ng), which is a neuron-specific protein that is enriched in the hippocampus and cortex (Represa et al., 1990; Gerendasy et al., 1994a,b). Two independent loss-of-function studies of Ng in mice highlight the importance of Ng in synaptic plasticity (Pak et al., 2000; Krucker et al., 2002). In this study, we explored the role of Ng in setting up synaptic plasticity balance by increasing Ng within CA1 hippocampal neurons. We have also explored whether Ng influences the ultrastructural localization of CaMKII and calcineurin using immunoelectron microscopy. Using a combination of molecular biology, electrophysiology, and electron microscopy, we have found that Ng shifts the frequency-response curve to the left without affecting CaMKII or calcineurin localization within dendritic spines. Interestingly, we have found that unlike CaMKII, which concentrates closer to the plasma membrane, calcineurin concentrates away from the plasma membrane. Taken together with our previous study showing that Ng concentrates CaM close to the plasma membrane highlights the significant role of Ng in setting the synaptic plasticity balance through the localized targeting of CaM within dendritic spines.

## Materials and Methods

### Animals and Hippocampal Slice Preparation

Young Sprague–Dawley rats (postnatal day 5 or 6) were purchased from Charles River Laboratories (Portage, MI, USA) and maintained on a 12 h light/dark cycle (lights off at 6:00 P.M.). Organotypic hippocampal slices were prepared as described previously (Gähwiler et al., 1997). All biosafety procedures and animal care protocols described here were approved by the Medical College of Wisconsin Institutional Animal Care and Use Committee and were performed in strict accordance with the Guidelines for Care and Use of Laboratory Animals of the National Institutes of Health.

### DNA Constructs and Expression

Ng was cloned by PCR from a commercial rat brain cDNA (Clontech, Mountain View, CA, USA). GFP-Ng was made with pEGFP plasmid and re-cloned into pSinRep5 (Invitrogen, Grand Island, NY, USA) for virus preparation as described (Zhong et al., 2009). After 5–7 days in culture, GFP-Ng was delivered into the slices using the Sindbis virus expression system, which is a replication-deficient, low-toxicity and neuron-specific system (Malinow et al., 1999).

### Post-embedding Immunogold Electron Microscopy

Organotypic hippocampal slices were fixed and processed for osmium-free post-embedding immunogold labeling as described earlier (Phend et al., 1995). Briefly, the CA1 region was carefully removed from the hippocampal slice and fixed with 0.1% picric acid, 1% paraformaldehyde, and 2.5% glutaraldehyde in 0.1 M phosphate buffer (pH 7.3) for 2 h at 4°C. After fixation, tissues were washed in 0.1 M maleate buffer (pH 6.0), treated with 1% tannic acid, 1% uranyl acetate, and 0.5% platinum chloride, followed by dehydration through a series of ethanol solutions. Tissues were then embedded in epoxy resins, sectioned and stained with 1% toluidine blue and 1% borax. CaMKII was labeled with an anti-CaMKIIα antibody (a generous gift from Dr. Johannes Hell, University of California, Davis, Davis, CA, USA) and an anti-mouse antibody coupled to 15-nm gold particles (Electron Microscopy Sciences, Hatfield, PA, USA). Calcineurin was labeled with an anti-calcineurin antibody (Sigma, St. Louis, MO, USA) and an anti-mouse antibody coupled to 10-nm gold particles (Electron Microscopy Sciences, Hatfield, PA, USA). Electron micrographs were obtained with a JOEL EM-2100 transmission electron microscope and an Orius SC 1000 CCD camera (JOEL, Peabody, MA, USA).

### Electrophysiology

Synaptic responses in organotypic slice cultures were evoked with two bipolar electrodes (FHC, Bowdoin, ME, USA) placed on the Schaffer collateral fibers between 300 and 500 μm of the recorded cells. The recording chamber was perfused with 119 mM NaCl, 2.5 mM KCl, 4 mM CaCl_2_, 4 mM MgCl_2_, 26 mM NaHCO_3_, 1 mM NaH_2_PO_4_, 11 mM glucose, 0.1 mM picrotoxin and 2 μM 2-chloroadenosine, at pH 7.4, and gassed with 5% CO_2_, 95% O_2_. Patch recording pipettes (3–6 MΩ) were filled with 115 mM cesium methanesulfonate, 20 mM CsCl, 10 mM HEPES, 2.5 mM MgCl_2_, 4 mM Na_2_ATP, 0.4 mM Na_3_GTP, 10 mM sodium phosphocreatine and 0.6 mM EGTA, at pH 7.25. LTD was induced by pairing 1 Hz presynaptic stimulation (500 pulses) with −40 mV postsynaptic depolarization. Voltage-clamp whole-cell recordings were acquired with a Multiclamp 700A amplifier (Axon Instruments, Union City, CA, USA).

### Statistical Analysis

Comparison of normalized average steady-state AMPA receptor-mediated responses between control uninfected neurons and Ng-infected neurons were carried out using unpaired *t*-tests. A comparison of cumulative distributions was carried out with the Kolmogorov–Smirnov test. Comparisons of the distribution of calcineurin or CaMKII between control and Ng-infected conditions were achieved using Chi-squared test. Values were considered significantly different if *p* ≤ 0.05, and marked with an asterisk. Error bars represent standard error of the mean in all figures.

## Results

### Ng Lowers LTD Expression

**Figure 1:**
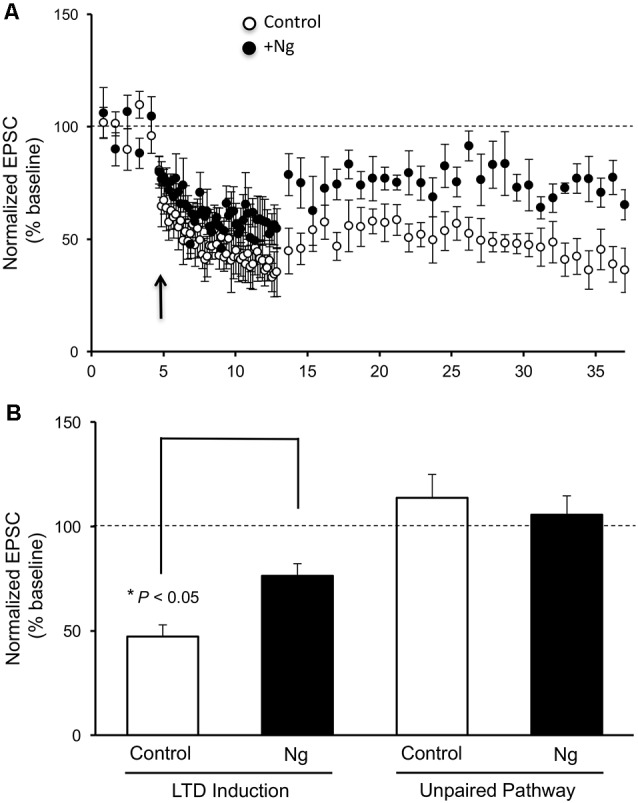
Neurogranin (Ng) decreases long-term depression (LTD) expression in CA1 hippocampal pyramidal neurons. **(A)** LTD was induced by pairing 1-Hz presynaptic stimulation (500 pulses) with −40 mV postsynaptic depolarization (indicated with an arrow) in neurons expressing GFP-Ng (black circles, *n* = 7) and control uninfected neurons (open circles, *n* = 8). **(B)** Normalized average steady-state AMPAR-mediated responses (between 25–37 min) in unpaired (control pathway) and paired (LTD pathway) pathways for uninfected neurons and those expressing GFP-Ng. Pairing significantly decreased AMPAR-mediated responses in both groups. Neurons expressing GFP-Ng showed decreased expression of LTD, compared to control neurons (*p* < 0.05).

Despite the significant role of Ng in learning and memory, the relevance of having more Ng in neurons on LTD induction has never been evaluated. To evaluate the role of Ng in LTD expression, we expressed Ng in organotypic hippocampal cultures and used whole-cell recordings from Ng-expressing and uninfected neurons under voltage-clamp configuration. As shown in Figure 1, Ng expression significantly decreased LTD expression as compared to control neurons.

### Neurogranin Regulates Metaplasticity at CA1 Hippocampal Synapses

**Figure 2:**
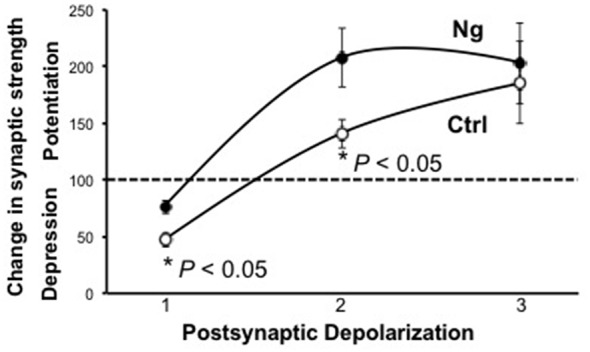
Ng regulates metaplasticity at CA1 hippocampal synapses. The graph represents experimental data from control and Ng-expressing neurons from organotypic hippocampal slices. All three protocols used were pairing protocols where presynaptic stimulation is paired with postsynaptic depolarization. Protocol #1:1 Hz stimulation (500 pulses) paired with −40 mV depolarization. Protocol #2:3 Hz stimulation (300 pulses) paired with −20 mV depolarization (Zhong and Gerges, 2012). The time-course of this experiment has been shown previously (Zhong and Gerges, 2012) Protocol #3:3 Hz stimulation (300 pulses) paired with 0 mV postsynaptic depolarization (Zhong et al., 2009). The time-course of this experiment has been shown previously (Zhong et al., 2009).

Metaplasticity refers to the sensitivity to change the threshold of LTP and LTD. On a molecular level, only a few molecules have shown such an effect on the synaptic plasticity threshold between LTP and LTD, such as CaMKII and postsynaptic density (PSD)95. We wished to examine the role of Ng in metaplasticity regulation. We have previously shown that Ng facilitates LTP (Zhong and Gerges, 2010, 2012). In the current study, we show Ng depresses LTD (Figure 1). To this end, we have plotted the steady-state AMPAR-mediated responses from our two previously published protocols that we used to induce LTP (Zhong et al., 2009; Zhong and Gerges, 2012) along with the protocol that we used for the current study to induce LTD. Figure 2 shows that Ng expression in CA1 hippocampal neurons results in a left shift. These data indicate that Ng regulates the metaplasticity at CA1 hippocampal neurons by favoring the induction of LTP and lowering that of LTD.

### Ng Does Not Change the Ultrastructural Localization of CaMKII

**Figure 3:**
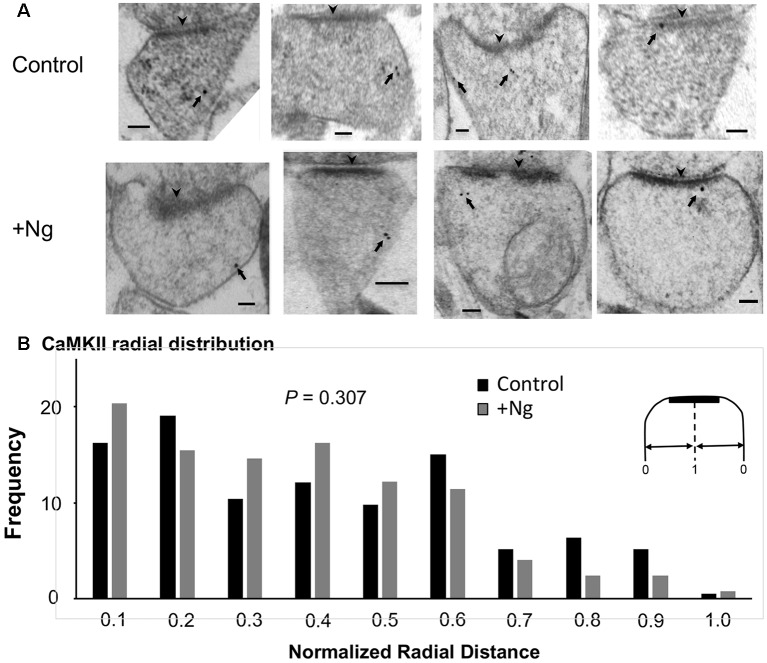
Ng does not change the ultrastructural localization of calmodulin (CaM)-dependent protein Kinase II (CaMKII). **(A)** Representative micrographs of immunogold-EM for endogenous CaMKII in dendritic spines of the CA1 area for control and Ng-overexpressing neurons. The black arrowheads point to the postsynaptic density (PSD). Arrows indicate anti-CaMKII immunogold particles. Scale bar, 100 nm. **(B)** Frequency histogram in control (*n* = 192) and Ng-overexpressing (*n* = 131) neurons. The distance of each gold particle was normalized (x-axis) to the corresponding radius of the spine measured through that particle. Ng does not change CaMKII radial distribution.

We have previously shown that Ng, CaM and CaMKII are not randomly distributed within dendritic spines and concentrate close to the plasma membrane (Zhong et al., 2009; Zhong and Gerges, 2012). Importantly, using post-embedding immunogold labeling electron microscopy, we have shown that increasing Ng levels targets more CaM close to the plasma membrane (Zhong and Gerges, 2012). Since Ng-mediated facilitation of synaptic plasticity is mediated by CaMKII, we wished to test the effect of increasing Ng on CaMKII distribution within the spine. To achieve this goal, we used post-embedding anti-CaMKII immunogold labeling on the synaptic region of the CA1 stratum radiatum of organotypic hippocampal slices overexpressing Ng, as previously described (Kaleka et al., 2012). To quantitatively assess the ultrastructural localization of CaMKII postsynaptically, we analyzed the distribution in a way similar to the one described previously (Zhong et al., 2009; Zhong and Gerges, 2012). Briefly, to assess the radial distribution, the shortest distance of each gold particle to the plasma membrane was measured and then normalized to the radius of the spine. Quantitative examination of CaMKII distribution shows that Ng overexpression did not change the radial distribution of CaMKII within dendritic spines (Figures 3A,B). Thus, while the overexpression of Ng targets more CaM close to the plasma membrane (Zhong and Gerges, 2012), it does not change CaMKII distribution.

### Ng Does Not Change the Ultrastructural Localization of Calcineurin

**Figure 4:**
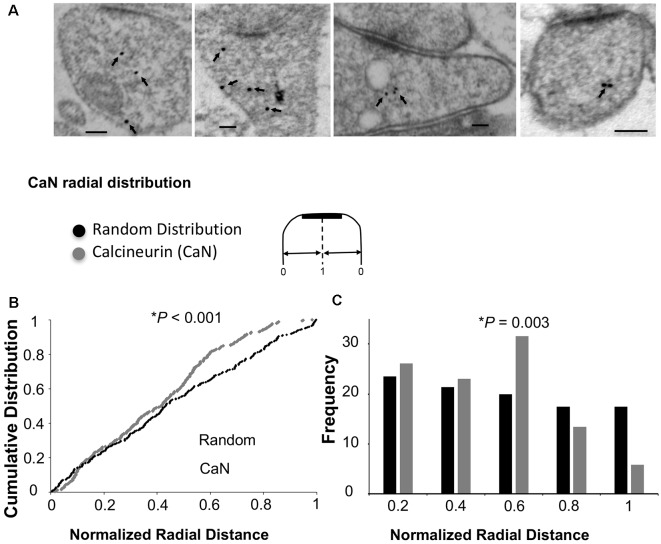
Ultrastructural localization of calcineurin. **(A)** Representative micrographs of immunogold-EM for calcineurin (CaN). **(B)** Cumulative probabilities of normalized radial distance. Normalized radial distribution was measured in the same way as described in Figure 3. The distribution of calcineurin within the spine (*n* = 291) is significantly different from a theoretical random distribution (Kolmogorov–Smirnov test, *p* < 0.001). **(C)** Frequency histogram for the data presented in **(B)** also showing that calcineurin distribution is significantly different from a theoretical random distribution using Chi-square. Interestingly, the highest fraction of labeling for calcineurin is not close to the plasma membrane but rather closer to the center of the spine (0.6 of the radial distance).

As mentioned above, Ca^2+^/CaM-dependent enzymes are essential for the bidirectional balance between LTP and LTD. While CaMKII is required for LTP, calcineurin is required for LTD. Since changing the ultrastructural localization of calcineurin could result in a change in the probability of its activation, we wished to test whether the overexpression of Ng changes the ultrastructural localization of calcineurin. To test this possibility, we first wished to understand the precise ultrastructural localization of endogenous calcineurin within dendritic spines. As shown in Figures 4A,B, calcineurin distribution is not random within dendritic spines. Interestingly, the highest frequency of calcineurin labeling was not found close to the plasma membrane (Figure 4C).

**Figure 5:**
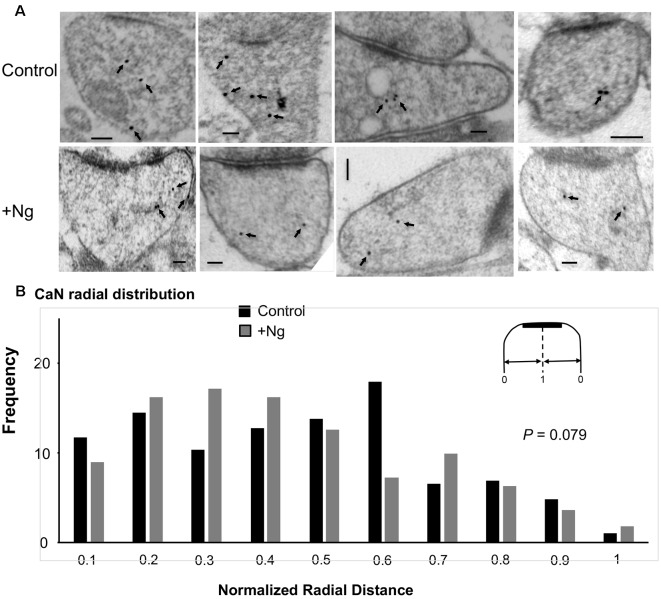
Ng does not change the ultrastructural localization of calcineurin. **(A)** Representative micrographs of immunogold-EM for calcineurin in control (same as of Figure 4) and Ng-overexpressing neurons. Scale bar, 100 nm. **(B)** Frequency histogram of the normalized radial distance of calcineurin particles. Ng does not change calcineurin radial distribution.

To investigate whether Ng influences calcineurin localization, we performed similar immunogold-EM labeling for calcineurin in tissues overexpressing Ng. Ng did not change the ultrastructural localization of calcineurin (Figure 5).

### Radial Distribution of Endogenous Ng, CaM, CaMKII and Calcineurin

**Figure 6:**
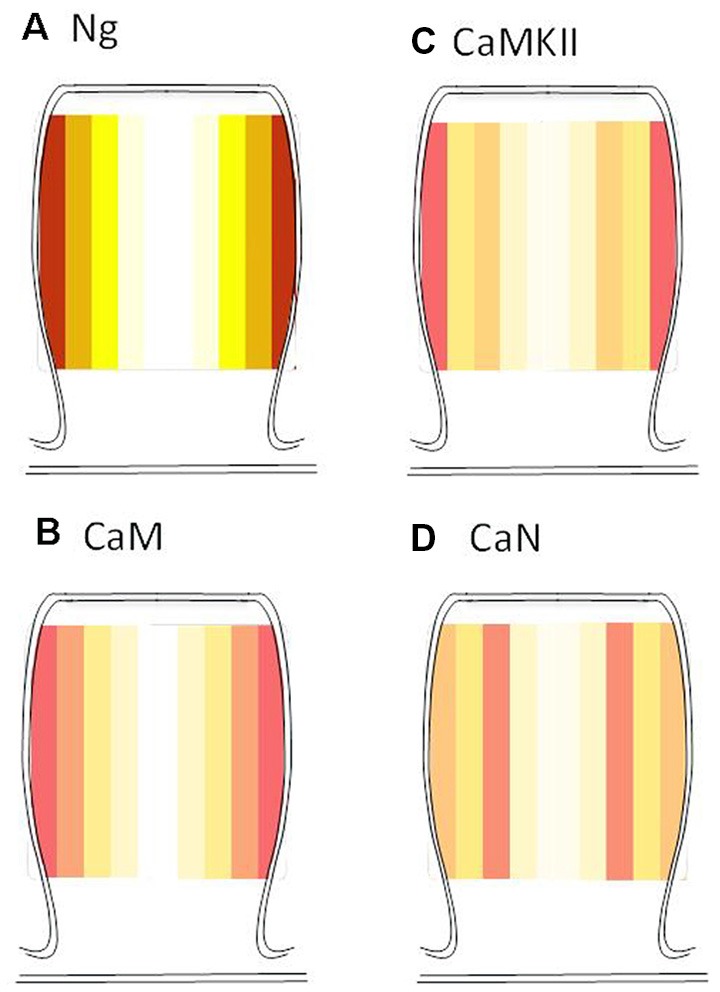
Radial distribution of endogenous Ng, CaM, CaMKII, and calcineurin. Heat maps were generated based on the actual radial distribution of endogenous Ng **(A)**, CaM **(B)**, CaMKII **(C)** and calcineurin **(D)** from immuno-EM labeling. Note that while CaMKII, CaM, and Ng have their highest concentration close to the plasma membrane, calcineurin has its highest concentration away from the plasma membrane of the dendritic spines. Note: Ng and CaM heat maps were generated by re-analyzing immunoEM data from Zhong et al. (2009) and Zhong and Gerges (2012), respectively.

While Ng did not change the ultrastructural localization of CaMKII or calcineurin, Ng does target CaM close to the plasma membrane (Zhong and Gerges, 2012). A closer look at the radial distribution indicates that while Ng, CaM, and CaMKII have a similar radial distribution (i.e., the highest concentration close to the plasma membrane), calcineurin distribution is distinct. Figure 6 shows the heat maps for the radial distribution of endogenous Ng (Zhong et al., 2009), CaM (Zhong and Gerges, 2012), CaMKII and calcineurin. These data suggest that Ng targeting of CaM closer to the plasma membrane within dendritic spines favors the activation of CaMKII at the expense of calcineurin.

## Discussion

Neuronal activity changes synaptic transmission. Long-lasting changes in synaptic transmission/strength are widely thought of as the cellular mechanisms of learning and memory. LTP and LTD are the most-studied forms of synaptic plasticity. Metaplasticity, a term that was coined by Abraham and Bear (Abraham and Bear, 1996), refers to the sensitivity to change the threshold of LTP and LTD. Both LTP and LTD induction depends on the NMDAR-mediated Calcium rise within dendritic spines and the subsequent activation of Ca/CaM-dependent enzymes. Thus the threshold of potentiation/depression (and hence metaplasticity) is dependent on the decision of CaM to either activate CaMKII (and hence the induction of LTP) or to activate calcineurin (and hence the induction of LTD). In this study, we show that Ng, a CaM-binding protein, regulates metaplasticity at CA1 hippocampal neurons.

It has been postulated that the differential affinity of CaM CaMKII and calcineurin is the sole determinant of CaM fate whether to activate CaMKII or calcineurin and subsequently induce LTP or LTD, respectively. The current study investigates the possible importance of the relative ultrastructural localization of Ng, CaM, CaMKII and calcineurin as a factor in regulating metaplasticity. While increased Ng expression does not change the ultrastructural localization of CaMKII or calcineurin, we have shown previously that Ng does target more CaM close to the plasma membrane within dendritic spines (Zhong and Gerges, 2012). In this study, calcineurin distribution reveals that the highest concentration of calcineurin does not lie closer to the plasma membrane, unlike Ng, CaM or CaMKII. Thus the ability of Ng to target more CaM close to the plasma membrane (where there is more CaMKII) may facilitate its activation and therefore, may facilitate LTP at the expense of LTD (i.e., produce a left-shift in the frequency-response curve).

Two independent loss-of-function studies of Ng in mice highlight the important function of Ng in regulating synaptic plasticity (Pak et al., 2000; Krucker et al., 2002; Huang et al., 2004). However, these studies gave opposite results, most likely due to the methodology employed for knocking out Ng. Interestingly, the study that had a complete knockout of Ng (Huang et al., 2004) showed a right shift of frequency-response curve, complementing our data here showing that increasing Ng results in a left shift of the frequency-response curve (i.e., increasing Ng favors LTP at the expense of LTD). Our findings are also in agreement with a computational study showing the need for a higher Ng concentration in the spines to be able to induce LTP at the expense of LTD (Zhabotinsky et al., 2006). Furthermore, a recent study showed that the downregulation of Ng lowered the threshold of LTD expression (Han et al., 2017). It remains to be tested, however, whether Ng can affect subsequent plasticity induction.

While there is a long list of molecules (Sanes and Lichtman, 1999) that block LTP induction, it is hard to believe that all of the molecules in this list control the synaptic plasticity balance. In fact, only a few manipulations have been described to influence the balance between LTP and LTD. For example, stress shifts the balance to the right, i.e., toward depression (Kim and Yoon, 1998). On the other hand, neurogenesis produces a left shift, i.e., toward potentiation (Garcia, 2002). Pharmacologically, prior NMDAR activation has been found to produce a right shift (Mockett et al., 2002). Conversely, prior activation of metabotropic glutamate receptors produced a left shift (Rush et al., 2002).

On a molecular level, only a few molecules have shown such an effect on the synaptic plasticity threshold between LTP and LTD. For example, overexpression of PSD95 occludes LTP and enhances LTD (Stein et al., 2003; Ehrlich and Malinow, 2004). Similarly, expression of the constitutively active form of CaMKII occludes LTP and favors the expression of LTD (Mayford et al., 1995). It is important to highlight that the effects of Ng, unlike the constitutively active CaMKII, are dependent on activity and the activation of NMDAR. Thus while the expression of the constitutively active CaMKII impairs learning and memory, Ng expression is expected to enhance learning and memory. This is indeed the case with transgenic mice overexpressing Ng in the prefrontal cortex (Zhong et al., 2015). For example, these mice exhibit higher extinction learning, a task that is dependent on PFC plasticity. The significant role of Ng in enhancing learning and memory was further supported by a recent study showing that the upregulation of Ng enables durable context encoding (Jones et al., 2018).

Either the inability of hippocampal neurons to express LTD (Mills et al., 2014) or the enhanced expression of LTD (Nosyreva and Huber, 2006) is often associated with cognitive deficits. However, manipulations that result in a left shift in the frequency-response curve (i.e., reduction in LTD expression) may be beneficial to memory. For example, prior activation of metabotropic glutamate receptors is associated with reduced LTD and enhanced memory (Rudy and Matus-Amat, 2009). We have recently shown that overexpression of Ng also enhances memory (Zhong et al., 2015). It remains to be tested, however, whether the enhanced memory as a result of increased Ng is due to the facilitation of LTP *per se*, the reduction of LTD *per se*, or the shift in the synaptic plasticity balance (i.e., the regulation of metaplasticity).

**Figure 7:**
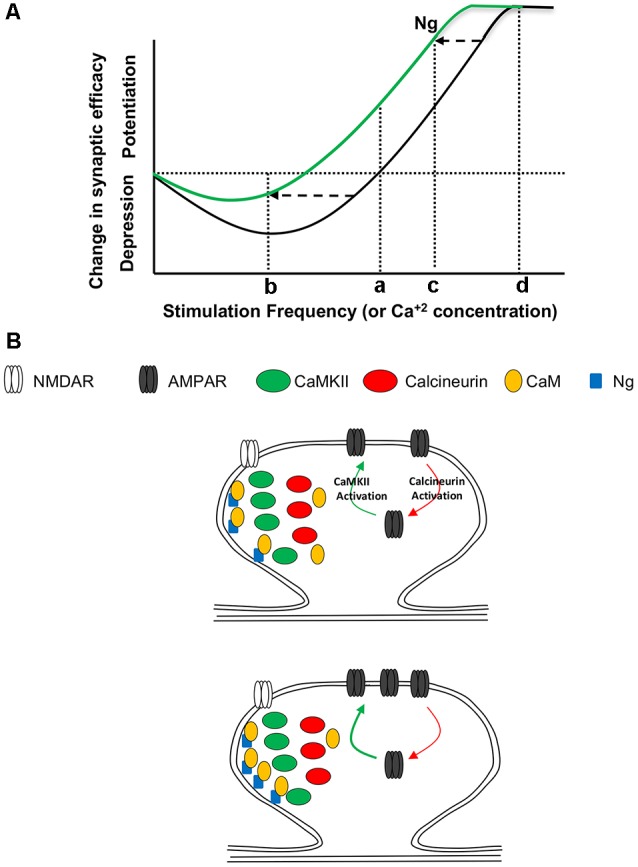
Ng and Metaplasticity. **(A)** Schematic diagram representing the changes in synaptic plasticity strength in response to various stimulation frequencies. Under normal conditions (black line), low-frequency stimulation (b) results in depression, while high-frequency stimulation (c) results in potentiation. Only a few things have been found to slide the whole curve; see “Discussion” section for details. This metaplasticity is similar to that described by the “sliding threshold” feature of the Bienenstock, Cooper and Munro model (BCM theory) of experience-dependent synaptic plasticity. This theoretical diagram resembles Figure 2. The various postsynaptic depolarization protocols used, represented by 1, 2 and 3 in Figure 2, are schematically equivalent to the stimulation frequencies b, c, and d, respectively. **(B)** A working model for Ng regulation of metaplasticity. (Top) While CaMKII, similar to Ng, concentrates closer to the plasma membrane, calcineurin concentrates away from the plasma membrane. Both CaMKII and calcineurin require Ca^2+^ and CaM to be activated. The activation of CaMKII results in the insertion of AMPA receptors and thus the expression of long-term potentiation (LTP). On the other hand, the activation of calcineurin results in the removal of AMPA receptors and thus the expression of LTD. (Bottom) Increasing Ng does not change the ultrastructural localization of CaMKII or calcineurin. Nonetheless, increasing Ng targets more CaM closer to the plasma membrane, where there is more CaMKII; thus increasing the chance of CaMKII activation, while decreasing the chance of CaN activation. This may explain, at least partly, why Ng shifts the synaptic plasticity curve to the left (i.e., regulating metaplasticity).

Our data support a model in which Ng regulates metaplasticity by shifting the frequency-response curve of synaptic plasticity curve to the left (i.e., facilitating LTP and lowering LTD; Figure 7). While Ng does not change the ultrastructural localization of CaMKII or calcineurin, Ng concentrates more CaM close to the plasma membrane of dendritic spines. Since CaMKII concentrates closer to the plasma membrane more than calcineurin does, Ng-mediated increases in CaM close to the plasma membrane may preferentially favor the potential activation of CaMKII over calcineurin. It is important to note that we have previously shown that Ng overexpression does not change NMDAR-mediated responses (Zhong et al., 2009), supporting a regulatory role of Ng in targeting CaM, rather than a global influencer role on the various signaling cascade.

## Data Availability Statement

The datasets generated for this study are available on request to the corresponding author.

## Ethics Statement

The animal study was reviewed and approved by the Medical College of Wisconsin Institutional Animal Care and Use Committee and were performed in strict accordance with the Guidelines for Care and Use of Laboratory Animals of the National Institutes of Health.

## Author Contributions

LZ and NG designed and performed the experiments, analyzed the data and wrote the manuscript.

## Conflict of Interest

The authors declare that the research was conducted in the absence of any commercial or financial relationships that could be construed as a potential conflict of interest.
